# Characterizing Micronutrient Status and Risk Factors among Late Adolescent and Young Women in Rural Pakistan: A Cross-Sectional Assessment of the MaPPS Trial

**DOI:** 10.3390/nu13041237

**Published:** 2021-04-09

**Authors:** Jo-Anna B. Baxter, Yaqub Wasan, Amjad Hussain, Sajid B. Soofi, Imran Ahmed, Zulfiqar A. Bhutta

**Affiliations:** 1 Department of Nutritional Sciences, University of Toronto, Toronto, ON M5S 1A8, Canada; joanna.baxter@mail.utoronto.ca; 2 Centre for Global Child Health, Hospital for Sick Children, Toronto, ON M5G 0A4, Canada; 3 Centre of Excellence in Women and Child Health, Aga Khan University, Karachi 74800, Pakistan; yaqub.wasan@aku.edu (Y.W.); amjad.hussain@aku.edu (A.H.); sajid.soofi@aku.edu (S.B.S.); imran.ahmed@aku.edu (I.A.); 4 Dalla Lana School of Public Health, University of Toronto, Toronto, ON M5T 3M7, Canada

**Keywords:** micronutrient status, adolescent nutrition, iron, vitamin A, vitamin D, anemia, determinants of health, risk factors

## Abstract

Nutritional deficiencies are a leading underlying risk factor contributing to the global burden of disease. In Pakistan, late adolescence is considered a nutritionally vulnerable period, as micronutrient requirements are increased to support maturation, and dietary staples are nutrient poor. However, there has been limited evaluation of micronutrient status beyond anemia and its determinants. Using cross-sectional data from late adolescent and young women (15–23 years) at enrolment in the Matiari emPowerment and Preconception Supplementation (MaPPS) Trial, we aimed to describe the prevalence of key micronutrient deficiencies of public health concern, and generate hierarchical models to examine associations with proxies for social determinants of health (SDoH). The prevalence of micronutrient deficiencies was high: 53.6% (95% confidence interval (CI): 53.0–54.3%) had anemia; 38.0% (95% CI: 36.4–39.6%) iron deficiency anemia; 31.8% (95% CI: 30.2–33.3%) vitamin A deficiency; and 81.1% (95% CI: 79.8–82.4%) vitamin D deficiency. At least one deficiency was experienced by 91.0% (95% CI: 90.1–92.0%). Few SDoH were maintained in the final hierarchical models, although those maintained were often related to socioeconomic status (e.g., education, occupation). To improve the micronutrient status of late adolescent and young women in Pakistan, a direct micronutrient intervention is warranted, and should be paired with broader poverty alleviation methods.

## 1. Introduction

Over the last decade, there has been increased attention to adolescence as a critical period for growth, learning, and neurobiological development [1]. Adequate nutrition, including sufficient vitamin and mineral intake, is essential given its underlying role in supporting the physiological process of maturation to adulthood; however, nutritional deficiencies disproportionately affect those in low- and middle-income settings [2]. While puberty delineates the start of adolescence, the point of entry into “adulthood” is less clearly defined, and is suggested by some to continue into the mid-twenties [3]. Notably, adolescent and young women are a particularly vulnerable population because of their increased nutritional requirements, possibility of discrimination due to gendered cultural norms, and potential for pregnancy [4].

Experiencing undernutrition during adolescence, including underweight, stunting, and micronutrient deficiencies, has been associated with poor cognitive and educational performance and decreased productivity [5]. Anemia, the most common micronutrient-related deficiency, is frequently attributed to lack of iron intake, but can also result from deficiencies in vitamins A, B12, or C; folate; or infectious diseases [6]. Other micronutrient deficiencies of widespread occurrence globally include iron deficiency (ID), vitamin A deficiency (VAD), and vitamin D deficiency (VDD). Within the ID spectrum, iron deficiency anemia (IDA) represents the severe end, and is estimated to be the largest cause of morbidity and mortality among adolescent girls globally [7]. As infection can also cause anemia, and inflammation can affect measures of iron deficiency, it is important to account for corresponding markers of inflammation such as C-reactive protein (CRP) or alpha-1-acid glycoprotein when reporting prevalence [8].

In Pakistan, the prevalence of anemia among adolescents and women of reproductive age (WRA) is high (i.e., >40%) [9]. The most recent National Nutrition Survey (NNS) reported that micronutrient deficiencies are widely experienced by married WRA, including IDA (18.2%), VAD (27.3%), and VDD (79.7%). Understanding how social determinants of health (SDoH) relate to micronutrient deficiencies could help to better frame programmatic actions and interventions to address the observed deficiencies among late adolescent and young women, as SDoH are fundamental social and economic factors that influence health and well-being [10].

Among unmarried and married, non-pregnant late adolescent and young women (15 to 23 years) living in rural Pakistan, we aimed to estimate the prevalence of key micronutrient deficiencies of public health interest; generate hierarchical models to examine the association between SDoH and the respective micronutrient deficiencies among late adolescent girls (15 to 19 years); and suggest potential programmatic actions to address the current challenges.

## 2. Materials and Methods

### 2.1. Conceptual Framework

**Figure 1:**
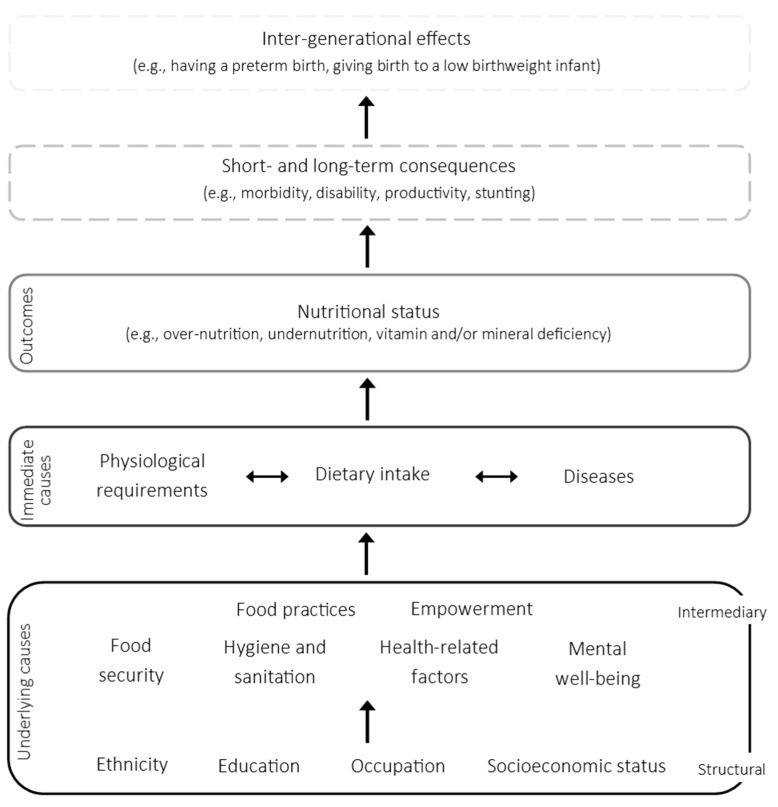
Conceptual framework of social determinants of health (SDoH) affecting the nutritional status of adolescent girls and their downstream sequelae.

We utilized a conceptual framework to serve as an overarching guide for how SDoH can affect the components that underlie nutritional status among females, as well as the downstream consequences (Figure 1). The structure was adapted from the UNICEF conceptual framework on the causes of malnutrition and informed by the World Health Organization (WHO) conceptual framework on SDoH [11,12].

### 2.2. Study Participants

We used cross-sectional data from the Matiari emPowerment and Preconception Supplementation (MaPPS) Trial, collected at enrolment, to perform all analyses. Details on the objectives, methodology, and data collection have previously been published [13,14]. Briefly, the MaPPS Trial is a two-arm, cluster-randomized effectiveness trial of life-skills-building education and multiple micronutrient supplementation provided from preconception among adolescent and young women in rural Pakistan (ClinicalTrials.gov Identifier: NCT03287882). To be eligible for inclusion, adolescent and young women had to be between 15 and 23 years of age, non-pregnant, physically able to comply with multiple micronutrient supplementation, not participating in any other nutrition studies, and intending to remain in the study area. Following confirmation of eligibility, the purpose and voluntary nature of the trial was explained, and an invitation to participate was extended. Written informed consent was obtained, as well as assent from participants <16 years. More than one person per household could be enrolled in the general study. To further monitor micronutrient status, a subgroup of randomly selected adolescent participants (<19 years) was formed, with a target enrolment of 132 adolescents per cluster [13]. To account for seasonal fluctuations, participants were recruited from the end of June 2017 to July 2018, with rollout by pre-identified clusters (health facility, *n* = 26).

### 2.3. Data Collection

All participants completed a multi-module questionnaire, anthropometric measurements, and point-of-care hemoglobin (Hb) assessment. The questionnaire included sections on demographics, socioeconomics, reproductive health, general health and nutrition practices, and empowerment. Anthropometric measures, including height and weight, were collected in duplicate by two data collectors. We used pre-set allowable differences (height: <1.0 cm; weight: <0.5 kg), and in the case that two measurements did not meet the criteria, a third measurement was completed. The average of two acceptable measures is presented for each participant. Hb concentration was assessed via finger prick by data collectors, using the HemoCue^®^ Hb 301 System (HemoCue; Ängelholm, Sweden). Participants enrolled in the micronutrient subgroup additionally provided a 5 mL venous blood sample collected by a phlebotomist, of which two drops were used to assess Hb concentration.

### 2.4. Lab Methods

Following collection, blood samples were maintained at 2 to 8 °C and transported to the field office for further processing. Following centrifugation, the serum was separated from the red blood cells, and transferred to cryovials. Serum samples were stored at −80 °C until analysis at the Nutrition Research Lab (NRL) at the Aga Khan University (AKU), and assessed for markers of nutritional status, including iron (ferritin concentration), vitamin A (retinol concentration), vitamin D (25-hydroxyvitamin D (25(OH)D) concentration), and the inflammation marker CRP. Ferritin and CRP were assessed using the immunoturbidimetric assay method (Cobas C311 Analyzer, Roche Diagnostics; ferritin kit: FERR4 (Tina-quant Ferritin Gen.4, #04885317 190); CRP kit: CRPLX (C-Reactive Protein [Latex], #20764930 322)); vitamin A using quantitative-high performance liquid chromatography photodiode array detection (Agilent HPLC, 1200/1260 Infinity Series with UV/PDA detection; manual method); and vitamin D using electrochemiluminescence protein binding assay (Diasorin Analyzer, LIAISON; kit: LIAISON^®^ 25 OH Vitamin D TOTAL Assay, #310600) [14]. Inter-assay CV values were determined (Table S1).

### 2.5. Categorization of Nutritional Status (Dependent Variables)

The WHO-generated definitions were used to classify participants’ nutritional status. The cut-offs were applied to body mass index (BMI) measures, including underweight (<18.5 kg/m^2^), normal (18.5–24.9 kg/m^2^), overweight (25–29.9 kg/m^2^), and obese (≥30 kg/m^2^) [15]. The standard WHO cut-offs for the key micronutrient deficiencies were also applied. Severe, moderate, and mild anemia were defined as Hb concentrations of <8.0, 8.0–10.9, and 11.0–11.9 g/dL, respectively [16]. ID was defined, per age- and sex-specific specifications, as a ferritin concentration of <15 ng/mL in the absence of inflammation, or an adjusted ferritin concentration of <70 ng/mL in the presence of inflammation, measured via CRP as a concentration of >5.0 mg/L [8]. Participants were excluded from ID estimation if a measure of inflammation was not available due to insufficient blood sample. Participants with iron overload (i.e., ferritin > 150 µg/L [8]) were also excluded from all iron-related analyses, as the aim was to understand deficiency. IDA was classified as any form of anemia (i.e., Hb concentration < 12 g/dL) with ID [6]. The general WHO cut-off for VAD in populations, defined as a retinol concentration <0.7 µmol/L, was applied [17]. VDD was defined as a 25(OH)D concentration <20 ng/mL, which is based on the concentration of 25(OH)D generally considered inadequate for the maintenance of bone and overall health [18].

### 2.6. SDoH (Explanatory Variables)

Proxy indicators for SDoH hypothesized to underlie micronutrient status were derived from the modules in the questionnaire administered at enrolment. These questions were taken from the Pakistan Demographic Health Survey (PDHS; [19]) and several standardized assessment tools ([20,21,22]; Table S2). We used a principal component analysis to generate a variable for wealth quintile from factors related to home characteristics and household asset ownership [19]. Food security was assessed using the Household Food Insecurity Access Scale (HFIAS), and a dichotomous variable (food secure and food insecure) was generated [23]. Scores for self-efficacy were categorized into tertiles corresponding to low, moderate, and high. Participation in decision-making was also categorized into three levels (all decisions made by family, most decisions made by family, decisions made jointly with family or autonomously) by applying the previously validated Survey-based Women’s Empowerment (SWPER) index method [24] to five questions on decision-making participation (personal food consumption, personal health care, household food purchases, household food distribution, household purchases), adapted from the PDHS [19].

### 2.7. Statistical Analysis

All analyses were conducted with Stata, version 15.0 (StataCorp, College Station, TX, USA). Data were considered cross-sectionally because they were collected prior to administering the study intervention. To describe the study population, descriptive statistics were used, including means with standard deviations (SDs) and counts with proportions for continuous and categorical variables, respectively.

To test whether there was a difference in micronutrient deficiency prevalence (anemia, IDA, VAD, VDD) between anthropometric-based nutritional status categories (BMI categories and stunting), the chi-squared test was used.

We aimed to generate a hierarchical model to examine the association between respective micronutrient status indicators (Hb concentration, IDA, VAD, and VDD) and hypothesized explanatory variables (SDoH). For all analyses, the base for each SDoH explanatory variable was set as the category hypothesized to present the greatest nutritional vulnerability (Table S2). First, crude analyses were conducted to identify any potentially relevant SDoH for inclusion. All variables with a *p*-value of <0.20 were considered potentially relevant [25]. This was done using linear regression for Hb concentration, and logistic regression for IDA, VAD, and VDD.

Using the conceptual framework as a guide, the SDoH explanatory variables were grouped into four levels: (1) socioeconomic status (education, occupation, religion, and wealth quintile); (2) household and personal factors (food security, marital status, and previous pregnancy); (3) health and well-being (perception of own health; experience of depression-, anxiety-, and stress-like feelings); and (4) actions and practices (self-efficacy, participation in decision-making, skipping breakfast, and eating dinner with family). The variables were entered into the model, starting at the distal-most level, using an adapted approach for hierarchical modelling [26]. At each level of the hierarchy, a backward stepwise model-building approach was used to serially remove variables identified for possible inclusion and find a model that best explained the data variability. We explored the exclusion of a variable from the model when the *p*-value of a variable was ≥0.05 or the standard error (SE) was high. In the case that the removal of a variable altered any of the β-coefficients of the variables retained in the model by >10%, the removed variable was returned. The effect estimates presented for the final model correspond to that at the point of addition to the model. For IDA, VAD, and VDD, these are presented as odds ratios (ORs) with 95% confidence intervals (CIs). To account for the cluster-randomized design of the MaPPS Trial, we adjusted for clustering at the health facility (*n* = 26).

### 2.8. Ethics Approval

Ethics approval for the MaPPS Trial was obtained from the AKU Ethics Review Committee and the Research Ethics Board at the Hospital for Sick Children, as well as the National Bioethics Committee of Pakistan.

## 3. Results

### 3.1. Participant Characteristics

Due to the criteria for inclusion in the micronutrient status subgroup, participants enrolled in the subgroup were younger than those in the larger trial (Table 1). Compared to the broader study population, micronutrient status subgroup participants were less educated, more often employed outside the home as a laborer, and from a poorer wealth quintile (all *p* < 0.001). Fewer were married (11.6% versus 22.3%; *p* < 0.001) or had been pregnant (5.6% versus 13.7%; *p* < 0.001).

### 3.2. Nutritional Status Characteristics

Complete anthropometric and food insecurity data were available for all 25,447 participants enrolled in the MaPPS Trial, and Hb concentration data were available for 25,443 (4 participants refused). A total of 3461 adolescent girls were enrolled in the micronutrient status subgroup, although data varied by biomarker: 3441 for ferritin (14 had iron overload; data not obtained for 6 due to an insufficient sample for analysis); 3423 for vitamin A (38 insufficient sample); 3437 for vitamin D (24 insufficient sample); and 3457 for CRP (4 insufficient sample). IDA was determined for 3438 participants, and all micronutrient status measures were complete for 3382. 

**Figure 2:**
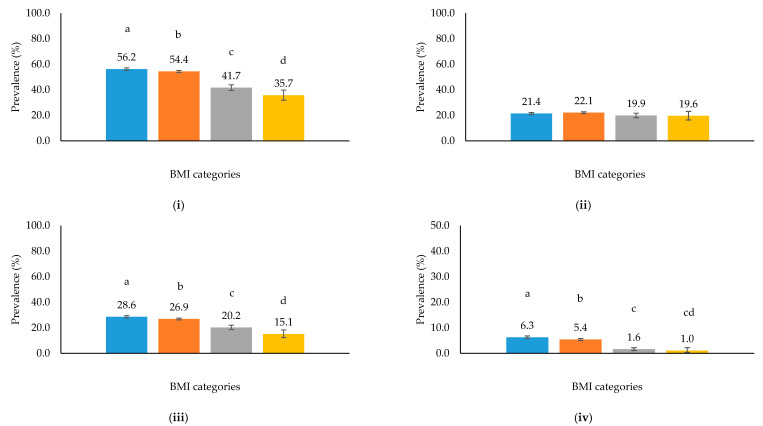
Prevalence of anemia by BMI categories (underweight: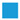, normal: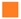, overweight: 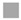, obese: 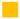) among MaPPS Trial participants (*n* = 25,447) at enrolment: (**i**) all types of anemia, (**ii**) mild anemia, (**iii**) moderate anemia, (**iv**) severe anemia. Bars with dissimilar letters are different (*p* < 0.05).

The prevalence of underweight, stunting, and household food insecurity was higher among those in the micronutrient status subgroup than the larger trial (42.0% versus 36.9%; 10.3% versus 9.2%; and 30.4% versus 25.0%, respectively; *p* < 0.05), although the prevalence of anemia was lower (47.6% versus 53.6%, respectively; *p* < 0.001; Table 2). Using all participant data, the prevalence of anemia differed by BMI categories for all forms of anemia (*p* < 0.001) except mild anemia (*p* = 0.07) (Figure 2i–iv). The prevalence of moderate and severe anemia differed between under- and normal-weight participants and with overweight and obese participants. More stunted participants were also anemic (59.3% versus 53.1%; *p* < 0.001).

**Figure 3:**
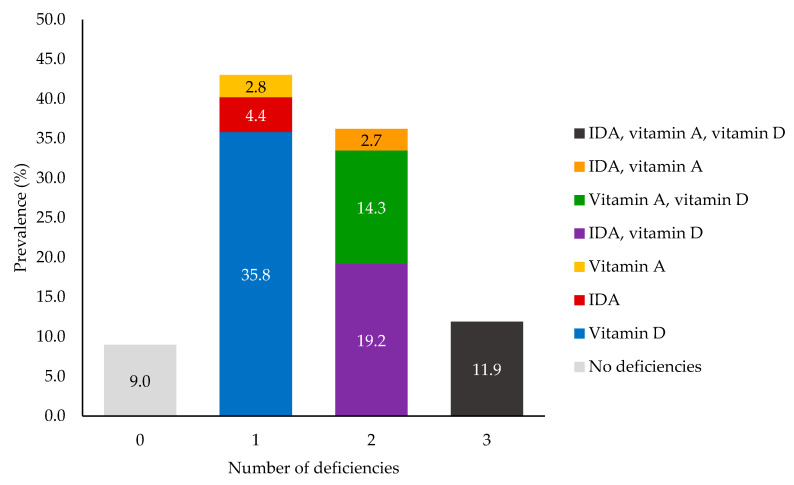
Number and type(s) of deficiencies experienced by late adolescent participants in the MaPPS Trial subgroup (*n* = 3382).

Among the adolescent girls in the micronutrient status subgroup, the prevalence of ID was high, at 65.9% (95% CI: 64.3–67.4%), and 38.0% (95% CI: 36.4–39.6%) of participants had IDA. Of those with anemia (*n* = 1649), 79.2% also had ID; however, of the participants with ID (*n* = 2264), 42.9% did not have anemia. The burden of acute inflammation was low (4.9%). VAD and VDD were found to affect 31.8% (95% CI: 30.2–33.3%) and 81.1% (95% CI: 79.8–82.4%) of participants, respectively. Of those with VAD, 4.7% of cases were severe, and 36.2% of those with anemia had concurrent VAD. When considering IDA, VAD, and VDD collectively, only 9.0% of participants had no deficiencies, yet 11.9% experienced all three forms of deficiency (Figure 3).

There was no difference in the prevalence of ID, VAD, or experience of ≥2 deficiencies between BMI categories (*p* = 0.22, 0.66, and 0.23, respectively), although compared to those who were overweight or obese, those who were underweight and normal weight had a higher prevalence of IDA (39.2% (underweight) versus 38.9% (normal) versus 24.2% (overweight or obese); *p* < 0.001) and experiencing ≥2 deficiencies (48.5% versus 48.3% versus 42.5%; *p* < 0.001), and a lower prevalence of VDD (78.3% versus 82.8% versus 86.3%; *p* = 0.001). With the exception of VDD, there was no difference in the prevalence of micronutrient deficiency based on stunting (ID: 61.4% versus 66.4%, *p* = 0.24; IDA: 40.9% versus 37.7%, *p* = 0.06; VAD: 33.5% versus 31.6%, *p* = 0.46; ≥2 deficiencies: 48.1% versus 48.0%; *p* = 0.98). The prevalence of VDD was lower among those who were stunted (74.4% versus 81.9%, *p* = 0.001).

### 3.3. SDoH and Micronutrient Status Hierarchical Model Generation

Because Hb concentration was determined for all participants enrolled in the trial, and because there was no difference in measures between adolescents (15–18.9 years) and young women (19–23 years; 11.5 ± 1.9 g/dL; *p* = 1.0), all data were considered to generate the hierarchical model. For the hierarchical models for IDA, VAD, and VDD, data were only collected among participants in the micronutrient status subgroup (15 to 18.9 years).

#### 3.3.1. Hb Concentration

Among the determinants from level 1, there was evidence that each variable (education level, occupation, religion, and wealth quintile) was associated with Hb concentration in the crude analyses (*p* < 0.001 for all values; Table 3). From level 2, having been pregnant and experiencing food security were associated with Hb concentration in the crude analysis (*p* < 0.05 and *p* < 0.001, respectively). Among level 3 variables, there was evidence from the crude analyses that Hb concentration was associated with perception of one’s health (*p* < 0.001) and experiencing depression-like or anxiety-like feelings (both *p* = 0.002). Finally, among level 4 variables, the crude analyses suggested there was evidence for an association between Hb concentration and self-efficacy and decision-making autonomy (both *p* < 0.001), and skipping breakfast also met the criteria for consideration in the multivariable model (*p* = 0.09).

After fitting the hierarchical model, variables that were maintained included level of education (*p* < 0.001), occupation (*p* < 0.001), wealth quintile (*p* < 0.001), having been pregnant (*p* < 0.001), and self-perception of one’s health (*p* = 0.001; Table 3). The significance of the final model was *p* < 0.0001. At level 1, the SE for religion was large, and its serial removal did not affect the other variables in the model. At level 2, the β-coefficient was small and SE large for food security, and the other variables in the model were not affected by its removal. At level 3, the association with experiencing depression-like and anxiety-like feelings was not maintained, and their SEs were large; thus, they were not retained. Similarly, at level 4, the association between self-efficacy, decision-making autonomy, and skipping breakfast with Hb concentration did not remain, and their SEs were large upon addition to the multivariable model. 

#### 3.3.2. IDA

Only the variable for education level was found to be associated with IDA in both the crude (*p* = 0.001) and adjusted models (*p* < 0.001; Table 4). Specifically, those with any education were found to have significantly lower odds of IDA compared to those with no education (primary education: OR: 0.82 (95% CI: 0.71 to 0.96); secondary education or higher: OR: 0.74 (95% CI: 0.63 to 0.87)). In the crude analyses, wealth quintile (*p* = 0.03) was found to be associated with having IDA, and occupation (*p* = 0.08), household food insecurity (*p* = 0.09), one’s perception of their own health (*p* = 0.14), self-efficacy (*p* = 0.10), and contribution to decision-making (*p* = 0.05) met the criteria to qualify for inclusion in the multivariable model. However, upon inclusion, the association of these variables with IDA did not remain and they were serially removed.

#### 3.3.3. Vitamin A Deficiency

No variables were maintained in the final model for VAD (Table 4). There was some evidence that religion (*p* = 0.03), wealth quintile (*p* = 0.007), household food insecurity (*p* = 0.04), experiencing depression-like feelings (*p* = 0.003), self-efficacy (*p* = 0.03), contribution to decision-making (*p* = 0.03), and eating meals with family (*p* = 0.001) were associated with VAD from the crude analyses. Additionally, marital status (*p* = 0.07), having had a previous pregnancy (*p* = 0.18), and self perception of one’s health (*p* = 0.16) met the criteria to qualify for inclusion in the model for VAD. However, after adjusting for clustering unit, no variables remained associated with VAD.

#### 3.3.4. Vitamin D Deficiency

For VDD, the variables maintained in the final hierarchical model included two variables from level 1 (education level and occupation) and one variable from level 3 (perception of own health; *p*-value for model <0.0001; Table 4). There was evidence that the odds of VDD increased for all occupations compared to performing unskilled manual labor. There was some evidence that education level was associated with increased odds of VDD—particularly among those with secondary education or more compared to those with no education (OR: 1.67 (95% CI: 1.17 to 2.38)). When explored, the removal of education level from the model affected the effect estimate for occupation by >10%, suggesting its role as a confounder. For perception of health, there was evidence that those who reported good or excellent health were at lower odds of VDD compared to those who reported poor or fair health. Variables that met the criteria for inclusion in the model included religion (*p* < 0.001); wealth quintile (*p* < 0.001); food insecurity (*p* < 0.001); experience of depression-, anxiety-, and stress-like feelings (*p* = 0.10, 0.02, and 0.02, respectively); self-efficacy (*p* = 0.15); contribution to decision-making (*p* = 0.03); and eating meals with family (*p* = 0.09). However, these were ultimately serially removed because their association with VDD was not maintained (food insecurity, depression- and anxiety-like feelings, self-efficacy, and eating meals with family), or their removal did not affect the β-coefficient of variables maintained in the model (religion, wealth quintile, experience of stress-like feelings, and contribution to decision-making).

## 4. Discussion

We documented a high prevalence of micronutrient deficiencies among late adolescent girls and young women in rural Pakistan. This is the first large-scale study in Pakistan to include micronutrient deficiencies among unmarried late adolescent girls and risk factors, although we did not find that marital status altered the observed prevalence. Ninety-one percent of the late adolescent girls experienced at least one of IDA, VAD, or VDD. From the generated multivariable models, few SDoH at the structural and intermediary levels were found to be associated with the respective micronutrient deficiencies of interest. Given the low prevalence of infections in the study population, consuming a nutrient-dense diet may present a more proximal burden to achieving micronutrient sufficiency. As there are very limited data available on the micronutrient status of adolescent girls in low- and middle-income countries, this study helps to fill this key data gap.

The burden of anemia observed among study participants (53.6%) corresponds to a “severe” level when applying the WHO population-level anemia classifications for public health significance [6]. Our findings are similar to the recent Pakistan NNS, which reported an anemia prevalence of 56.6% and 41.7% among adolescent girls (10–19 years) and married WRA (15–49 years), respectively [9]. The proportion of married WRA with severe anemia at the national level was lower than in our study (~1% versus ~5%, respectively), although the prevalence of food insecurity was lower in our study compared to the NNS (25.0% versus 41.7% in Sindh province). However, we assessed food security using differing tools [23,27]. Notably, non-pregnant adolescent and young women are at an increased risk for anemia due to the regular blood loss that occurs with menstruation [6].

In investigating anemia further using all participant data, we found the prevalence of any form of anemia was high, regardless of whether one was underweight, normal, or overweight. This is suggestive of a double burden of malnutrition. Because we had Hb measures at the individual level, we used Hb concentration to generate the hierarchical multivariable model. Predominantly structural SDoH variables were maintained in the final model, highlighting the importance of poverty. Several studies of the factors affecting anemia prevalence among adolescent girls have been conducted in India, and having higher education has consistently been suggested to be protective of anemia, which is consistent with our findings [28,29,30,31,32]. Among WRA in multiple countries, Wirth et al. [33] also found socioeconomic status was inversely associated with anemia prevalence.

Surprisingly, being an adolescent girl who had never been pregnant was found to be associated with having a lower Hb concentration. Previous studies of WRA have suggested that having short durations between pregnancies and/or a high number of pregnancies increases one’s risk for anemia due to excess demands [6]. The proportion of participants who had been pregnant in our study was low, and few had had more than one pregnancy. Anthropometric data also suggested some younger, nulligravida participants might still be growing. Our findings could reflect that nulligravida adolescent girls have poor dietary intake patterns, given the general finding that adolescents are prone to consuming nutrient-poor snack foods [34].

Among the late adolescent girls in the subgroup who had anemia, the majority were found to have concurrent ID. The difference in the prevalence of anemia between the subgroup and larger study likely reflects to the use of venous versus capillary blood, respectively, for assessing Hb concentration [35]. The prevalence of ID itself was high among subgroup participants (65.9%), although not all participants with ID had anemia. Notably, the prevalence of inflammation was low from CRP levels. Other studies have found that in countries with high infection rates, the proportion of anemia attributable to iron deficiency is lower [33]. Considering this information collectively, the provision of a supplement containing iron should be considered as a public health measure [36,37]. Dietary diversification and food fortification strategies could also be of benefit, although the magnitude of the problem meets the criteria for a targeted intervention.

We observed an IDA prevalence of 38.0% in this study, which is higher than the NNS estimates for WRA in Sindh Province (23.8%) [9]. This could reflect the lower age range of our participants. From the hierarchical modelling, while some variables were associated with IDA in the bivariate analyses, only education level was maintained post-adjustment for clustering. Given the direct and indirect costs of educating girls in this setting, those with higher education are more likely to be from wealthier families, and could consume more nutritious diets (particularly meat), although the variable for wealth quintile itself was not maintained in the model.

With respect to VAD, the prevalence of deficiency was high at 31.8% among the late adolescent subgroup participants. Among married WRA, the recent NNS reported that 27.4% experienced VAD [9]. This is an improvement from past national surveys [38]. Surprisingly, no SDoH variables were maintained in the multivariable model for VAD. As vitamin A is principally obtained from the diet, and we found low levels of inflammation, this could suggest the importance of diet to deficiency. As with iron, direct and indirect interventions could improve vitamin A intake in this setting. 

The prevalence of vitamin D deficiency in Pakistan is documented to be high. We found that 81.1% of the late adolescent subgroup participants had vitamin D deficiency. Among married WRA, the NNS found that VDD affected 79.7% [9]. Unlike iron and vitamin A, vitamin D is obtained primarily from the exposure of the skin to sun [18]. From early childhood, Pakistani girls usually wear clothing that covers much of their body, limiting sun exposure. Within the multivariable model, unskilled laborers were at less risk for VDD compared to all other occupations. Unskilled laborers commonly perform manual labor outdoors, and in this setting farming is predominant, suggesting increased sun exposure. Given the limited potential to obtain vitamin D from the diet, supplementation or fortification could serve to increase intake. 

Collectively, our findings suggest that there is much opportunity for the improvement of the micronutrient status of all late adolescent and young women in Pakistan. The Pakistan Cost of the Diet Analysis recently estimated that two out of three households in Pakistan are unable to afford a nutritionally adequate diet [39]. The availability of nutrient-rich foods was not a key barrier in obtaining a nutritious diet, as dietary requirements could be met using foods available in local markets, unless families were restricted by economic constraints (i.e., barriers to access). An “affordability gap” was identified among poor households, and without increasing income consumption patterns are suggested to be unlikely to change. In their assessment, vitamins and minerals that are obtained from predominantly animal-sourced foods (e.g., iron, vitamin B12, calcium) were particularly more difficult to access. Paired with the fact that adolescents are more prone to eat less-nutritious foods [34], this suggests a need to support dietary intake improvement measures.

There is a recognized need in Pakistan to focus on adolescent girls’ health and nutrition, and guidelines have been developed in accordance with WHO policy [40,41]. However, we would note that for such guidelines to produce a meaningful change, dedicated ownership by the government and formal policy generation will be critical. Recently, the Government of Pakistan released its nutritional strategy and operational plan for targeting adolescent malnutrition [42]. This is an important document for generating legislation, and it mirrors many of the recommendations discussed below—particularly the role of iron and folic acid supplementation, food fortification, and broader efforts around poverty alleviation strategies. At the time of writing, finalized guidelines for adolescent nutrition and supplementation in Pakistan are described to be released soon, and will include additional details on how monitoring and evaluation can be built into the implementation process, as well as assessment of coverage and quality. From the draft documents, we would note that since the high burden of the micronutrient deficiencies is not specific to underweight adolescent girls, as we have described, there may be a role for broadening micronutrient tablet provision.

Because the proportion of girls who are in school during late adolescence is low, and many never attended school, reach could be enhanced by offering parallel engagement through the community and schools. In communities, the existing Lady Health Worker (LHW) Programme should be leveraged. Within the LHW Programme, primary health services are offered via a cadre of female community health workers called LHWs [43]. Traditionally, this has targeted married women, for the purposes of reproductive health and pregnancy monitoring, and infants <5 years; however, it could be leveraged to reach unmarried adolescent and young women. To indirectly deal with the strains on micronutrient status, programming should be offered from early adolescence (i.e., 10 years) and aim to address stratifiers for poverty important to the setting (e.g., continuing with one’s education, delaying marriage and first pregnancy). Additionally, teaching appropriate nutritional practices could serve to improve nutrition knowledge, because this is noted to be limited in the Pakistani context [44].

Since the prevalence of anemia and specific micronutrient deficiencies is high in Pakistan, evidence-based interventions should be considered to directly address nutritional status. In accordance with the WHO guidelines, the criteria for iron supplementation among menstruating, non-pregnant women are met, as previously mentioned [36,37]. Given that several micronutrient deficiencies exist, both evidenced by our data and data collected at a national level in Pakistan [9], a multiple-micronutrient supplement may offer additional benefit. The fortification of staple foods could also serve to passively increase iron and vitamin A and D intake. Since 2016, implementing large-scale food fortification, including wheat flour (fortified with iron, folic acid, vitamin B12, and zinc) and edible oil and ghee (vitamin A and D), has been an increased focus of the Pakistani government with partners [45]. Wheat flour fortification, in particular, has been found to be cost effective in this setting, although requirements for impact include that fortification will need to be mandatory among manufacturers and abidance with compliance and fortifying standards ensured [46].

The present cross-sectional study is not without limitations. As there is dietary variability in the different provinces within Pakistan, the findings here may not reflect all settings, although the SDoH characteristics of those sampled were generally consistent with rural Pakistan. We did not assess markers for some micronutrients known to contribute to anemia (e.g., vitamin B12, folate), so we cannot attest to whether deficiencies in these micronutrients were experienced by study participants. In the previous cycle of the NNS, Soofi et al. [47] found that 52.4% and 50.8% of married WRA experienced vitamin B12 and folate deficiency, respectively. Similarly, we did not assess alpha-1-acid glycoprotein, a marker of chronic inflammation, so we could not complete some of the more sophisticated corrections for ID and inflammation suggested in the literature [48,49]. We would note that other studies of pregnant women and early adolescent boys and girls in this region in Pakistan have similarly suggested that the burden of inflammation is low [50,51]. We also did not assess a comprehensive set of biomarkers to understand iron deficiency (e.g., hepcidin, transferrin receptor protein), nor did we assess hemoglobinopathies. At this time hemoglobinopathies are not considered to exist widely in Pakistan, although they are of growing concern in several Southeast Asian countries [52]. Finally, because of the unique cultural practices and dietary patterns of adolescent and young women in Pakistan, our findings may not be generalizable to the settings in other countries.

In terms of future research, we hope to better understand the effect of the provision of multiple-micronutrient supplements on anemia and micronutrient status among participants in the MaPPS Trial context.

## 5. Conclusions

The adolescent period can be a vulnerable time for girls and young women, given the potential effect of gendered cultural norms that limit their access to education, nutritious foods, and other opportunities [4]. However, it has also been identified as a promising “window of opportunity” for nutritional intervention [53]. The observed prevalence of micronutrient deficiencies, found both in this study and from national estimates, highlights an important area for improvement among late adolescent and young women in Pakistan. There is a need for both direct and indirect nutrition interventions that are integrated and multi-sectoral, starting with strategies to address agency and empowerment through education, reducing early marriages and food poverty, as well as integration with school health and nutrition services for girls and food fortification programs [54].

## Figures and Tables

**Table 1:** Descriptive characteristics among all participants at enrolment in the Matiari emPowerment and Preconception Supplementation (MaPPS) Trial (*n* = 25,447) and the micronutrient status subgroup (*n* = 3461).

Characteristic	All Participants	Micronutrient Status Subgroup	*p*-Value
Age (years), mean ± SD	18.8 ± 2.3	17.2 ± 1.2	<0.001
Highest level of education completed, *n* (%)			
None	11,384 (44.7)	1615 (46.7)	<0.001
Primary	6063 (23.8)	842 (24.3)	
Secondary or higher	8000 (31.5)	1004 (29.0)	
Employed outside the home, *n* (%)	9620 (38.1)	1391 (40.2)	<0.001
Muslim, *n* (%)	23,045 (90.6)	3116 (90.0)	<0.001
Wealth quintile, *n* (%)			
Poorest	4549 (17.9)	680 (19.6)	<0.001
Poor	4853 (19.1)	734 (21.2)	
Middle	5139 (20.2)	769 (22.2)	
Rich	5360 (21.1)	726 (21.0)	
Richest	5546 (21.8)	552 (15.9)	
Currently married ^1^, *n* (%)	5685 (22.3)	400 (11.6)	<0.001
Age when married husband (years), mean ± SD	16.9 ± 1.8	15.8 ± 1.1	<0.001
Has been pregnant ^2^, *n* (%)	3478 (13.7)	194 (5.6)	<0.001
Age at first pregnancy ^2^, mean ± SD	17.5 ± 1.6	16.5 ± 1.2	<0.001

^1^ 158 of all participants reported no longer being married (16 in the micronutrient status subgroup). ^2^ Asked only of ever-married women given cultural sensitivities.

**Table 2:** Nutritional-status-related characteristics among participants at enrolment in the MaPPS Trial (*n* = 25,447) and micronutrient status subgroup (*n* = 3461).

Characteristic	All Participants	Micronutrient Status Subgroup	*p*-Value
BMI categorization, *n* (%)			
Underweight (<18.5 kg/m^2^)	9396 (36.9)	1452 (42.0)	<0.001
Normal (18.5–24.9 kg/m^2^)	13,463 (52.9)	1770 (51.1)	
Overweight (25–29.9 kg/m^2^)	2011 (7.9)	203 (5.9)	
Obese (≥30 kg/m^2^)	577 (2.3)	36 (1.0)	
Stunted (<145 cm), *n* (%)	2345 (9.2)	358 (10.3)	0.04
Food insecure, *n* (%)	6355 (25.0)	1052 (30.4)	<0.001
Hb concentration ^1^ (g/dL), mean ± SD	11.5 ± 2.0	11.8 ± 1.9	<0.001
Anemia status ^1^, *n* (%)			
Mild (11.9–11.0 g/dL)	5497 (21.6)	735 (21.2)	<0.001
Moderate (10.9–8.0 g/dL)	6798 (26.7)	752 (21.7)	
Severe (<8.0 g/dL)	1353 (5.3)	162 (4.7)	
Serum ferritin concentration ^2^ (µg/L), geometric mean (95% CI)	-	10.4 (10.1 to 10.8)	-
Iron deficiency ^3^, *n* (%)	-	2264 (65.9)	-
Iron deficiency anemia ^4^, *n* (%)	-	1307 (38.0)	-
Serum retinol concentration ^6^ (µmol/L), mean ± SD	-	0.96 ± 0.49	-
Vitamin A deficiency ^5^, *n* (%)	-	1092 (31.8)	-
Serum 25(OH)D concentration ^5^ (ng/mL), mean ± SD	-	14.9 ± 7.9	-
Vitamin D deficiency ^6^, *n* (%)	-	2777 (81.1)	-
Number of deficiencies ^7^, mean ± SD		1.5 ± 0.8	
CRP concentration, (mg/L), mean ± SD	-	1.42 ± 3.78	-
Acute inflammation ^8^, *n* (%)	-	171 (4.9)	-

^1^ Data not obtained for 4 participants in MaPPS Trial (*n* = 25,443). ^2^ Data not obtained for 6 participants, and participants with iron overload (serum ferritin > 150 µg/L; *n* = 14) were excluded (*n* = 3441). ^3^ Defined as serum ferritin < 15 µg/L without acute inflammation (C-reactive protein (CRP) < 5 mg/L), or serum ferritin < 70 µg/L with acute inflammation (CRP > 5 mg/L) (*n* = 3438). ^4^ Defined as iron deficiency and anemia (hemoglobin (Hb) < 12 g/dL; *n* = 3438). ^5^ Data not obtained for 24 participants (*n* = 3437). ^6^ Data not obtained for 38 participants (*n* = 3423). ^7^ Possible deficiencies included iron deficiency anemia (IDA), vitamin A deficiency, and vitamin D deficiency (*n* = 3382). ^8^ Defined as CRP >5 mg/L, data not obtained for 4 participants (*n* = 3457).

**Table 3:** Comparison of SDoH with Hb concentration among participants in the MaPPS Trial at enrolment (*n* = 25,443).

SDoH	*n* (%)	Hb Concentration (g/dL) Mean (95% CI)	Crude Analysis		Multivariable Adjusted Analysis	
			β-Coefficient (95% CI)	*p*-Value	β-Coefficient (95% CI)	*p*-Value
*Level 1: Socioeconomic status*						
Highest level of education				<0.001		<0.001
None	11,382 (44.7)	11.3 (11.3 to 11.4)	(reference)		(reference)	
Primary	6062 (23.8)	11.6 (11.5 to 11.6)	0.26 (0.20 to 0.32)		0.14 (0.08 to 0.20)	
Secondary or higher	7999 (31.4)	11.8 (11.8 to 11.9)	0.50 (0.44 to 0.55)		0.28 (0.21 to 0.35)	
Occupation				<0.001		<0.001
Unskilled manual labor	4458 (17.5)	11.2 (11.1 to 11.2)	(reference)		(reference)	
Skilled manual labor	5133 (20.2)	11.7 (11.6 to 11.7)	0.48 (0.41 to 0.56)		0.23 (0.15 to 0.32)	
Within the home	12,129 (47.7)	11.5 (11.5 to 11.6)	0.37 (0.31 to 0.44)		0.14 (0.07 to 0.21)	
Other	3723 (14.6)	11.8 (11.7 to 11.8)	0.62 (0.53 to 0.70)		0.12 (0.01 to 0.22)	
Religion				<0.001		-
Hindu	2401 (9.4)	11.4 (11.3 to 11.4)	(reference)		-	
Muslim	23,042 (90.6)	11.6 (11.5 to 11.6)	0.19 (0.11 to 0.27)		-	
Wealth quintile				<0.001		<0.001
Poorest	4548 (17.9)	11.2 (11.2 to 11.3)	(reference)		(reference)	
Poor	4853 (19.1)	11.4 (11.3 to 11.4)	0.11 (0.04 to 0.19)		0.03 (−0.05 to 0.11)	
Middle	5138 (20.2)	11.5 (11.4 to 11.6)	0.25 (0.18 to 0.33)		0.13 (0.05 to 0.21)	
Rich	5360 (21.1)	11.7 (11.6 to 11.7)	0.41 (0.33 to 0.48)		0.22 (0.14 to 0.30)	
Richest	5544 (21.8)	11.8 (11.8 to 11.9)	0.60 (0.53 to 0.68)		0.34 (0.24 to 0.43)	
*Level 2: Household and personal characteristics*						
Marital status				0.72		-
Married	5685 (22.3)	11.3 (11.3 to 11.4)	(reference)		-	
Unmarried	19,758 (77.7)	11.6 (11.6 to 11.6)	−0.01 (−0.07 to 0.05)		-	
Has been pregnant				0.05		<0.001
Yes	3478 (13.7)	11.5 (11.5 to 11.6)	(reference)		(reference)	
No	21,965 (86.3)	11.5 (11.5 to 11.6)	−0.07 (−0.14 to 0)		−0.15 (−0.22 to −0.08)	
Household food security status				<0.001		-
Food insecure	6354 (25.0)	11.6 (11.5 to 11.7)	(reference)		-	
Food secure	19,089 (75.0)	11.5 (11.5 to 11.6)	0.30 (0.24 to 0.35)		-	
*Level 3: Health and well-being characteristics*						
Perception of own health				<0.001		0.001
Poor or fair	2808 (11.0)	11.3 (11.3 to 11.4)	(reference)		(reference)	
Good	16,556 (65.1)	11.5 (11.5 to 11.6)	0.21 (0.13 to 0.29)		0.14 (0.06 to 0.21)	
Excellent	6079 (23.9)	11.6 (11.6 to 11.7)	0.28 (0.19 to 0.36)		0.17 (0.08 to 0.25)	
Experience of depression-like feelings				0.002		-
Severe or extremely severe	1223 (4.8)	11.5 (11.4 to 11.6)	(reference)		-	
Moderate	2652 (10.4)	11.4 (11.4 to 11.5)	−0.06 (−0.19 to 0.07)		-	
Mild	2158 (8.5)	11.5 (11.4 to 11.5)	−0.03 (−0.16 to 0.10)		-	
None	19,410 (76.3)	11.6 (11.5 to 11.6)	0.07 (−0.04 to 0.18)		-	
Experience of anxiety-like feelings				0.002		-
Severe or extremely severe	3313 (13.0)	11.5 (11.4 to 11.5)	(reference)		-	
Moderate	3637 (14.3)	11.5 (11.4 to 11.5)	0.02 (−0.07 to 0.11)		-	
Mild	1684 (6.6)	11.5 (11.4 to 11.6)	0.07 (−0.04 to 0.19)		-	
None	16,809 (66.1)	11.6 (11.5 to 11.6)	0.11 (0.04 to 0.19)		-	
Experience of stress-like feelings				0.55		-
Severe or extremely severe	895 (3.5)	11.5 (11.4 to 11.7)	(reference)		-	
Moderate	1356 (5.3)	11.5 (11.4 to 11.6)	−0.05 (−0.21 to 0.11)		-	
Mild	1506 (5.9)	11.5 (11.4 to 11.6)	−0.04 (−0.20 to 0.12)		-	
None	21,686 (85.2)	11.5 (11.5 to 11.6)	0.01 (−0.12 to 0.14)		-	
*Level 4: Actions and practices-related characteristics*						
Self-efficacy				<0.001		-
Low	8630 (33.9)	11.4 (11.4 to 11.5)	(reference)		-	
Moderate	12,868 (50.6)	11.6 (11.5 to 11.6)	0.16 (0.11 to 0.21)		-	
High	3945 (15.5)	11.6 (11.6 to 11.7)	0.22 (0.15 to 0.29)		-	
Decision-making autonomy				0.002		-
All decisions made by family	12,606 (49.5)	11.5 (11.5 to 11.5)	(reference)		-	
Most decisions made by family	8332 (32.7)	11.6 (11.5 to 11.6)	0.06 (0.003 to 0.11)		-	
Jointly made with family or autonomously	4505 (17.7)	11.6 (11.6 to 11.7)	0.11 (0.05 to 0.18)		-	
Skipping breakfast				0.09		-
Skips breakfast	7608 (29.9)	11.5 (11.5 to 11.5)	(reference)		-	
Eats breakfast	17,835 (70.1)	11.5 (11.5 to 11.6)	0.04 (−0.01 to 0.10)		-	
Eating dinner with family				0.68		-
Never	6820 (26.8)	11.6 (11.5 to 11.6)	(reference)		-	
Sometimes	3489 (13.7)	11.5 (11.5 to 11.6)	−0.03 (−0.11 to 0.05)		-	
Everyday	15,134 (59.5)	11.5 (11.5 to 11.6)	−0.02 (−0.07 to 0.03)		-	

**Table 4:** Comparison of SDoH with IDA, vitamin A deficiency (VAD), and vitamin D deficiency (VDD) among adolescent girls enrolled in the MaPPS Trial micronutrient status subgroup.

SDoH	IDA (*n* = 3438)				Vitamin A Deficiency (*n* = 3437)				Vitamin D Deficiency (*n* = 3423)			
	Crude		Multivariable Adjusted		Crude		Multivariable Adjusted		Crude		Multivariable Adjusted	
	OR (95% CI)	*p*-Value	OR (95% CI)	*p*-Value	OR (95% CI)	*p*-Value	OR (95% CI)	*p*-Value	OR (95% CI)	*p*-Value	OR (95% CI)	*p*-Value
*Level 1: Structural factors—socioeconomic status*												
Highest level of education												
None	(base)	-	(base)	-	(base)	-	-	-	(base)	-	(base)	-
Primary	0.82 (0.69 to 0.98)	0.03	0.82 (0.71 to 0.96)	0.01	0.85 (0.71 to 1.02)	0.08	-	-	1.43 (1.16 to 1.77)	0.001	1.28 (0.97 to 1.71)	0.08
Secondary or higher	0.74 (0.63 to 0.87)	<0.001	0.74 (0.63 to 0.87)	<0.001	0.93 (0.79 to 1.10)	0.42	-	-	2.19 (1.75 to 2.73)	<0.001	1.67 (1.17 to 2.38)	0.01
Occupation												
Unskilled manual labor	(base)	-	-	-	(base)	-	-	-	(base)	-	(base)	-
Skilled manual labor	0.87 (0.70 to 1.08)	0.22	-	-	1.01 (0.81 to 1.27)	0.91	-	-	2.78 (2.11 to 3.67)	<0.001	2.56 (1.70 to 3.87)	<0.001
Within the home	0.88 (0.73 to 1.05)	0.16	-	-	1.07 (0.88 to 1.29)	0.50	-	-	1.93 (1.57 to 2.37)	<0.001	1.74 (1.40 to 2.16)	<0.001
Other	0.74 (0.59 to 0.93)	0.01	-	-	1.08 (0.85 to 1.38)	0.51	-	-	3.34 (2.46 to 4.54)	<0.001	2.19 (1.49 to 3.24)	<0.001
Religion												
Hindu	(base)	-	-	-	(base)	-	-	-	(base)	-	-	-
Muslim	0.98 (0.78 to 1.23)	0.85	-	-	1.31 (1.02 to 1.68)	0.04	-	-	1.79 (1.39 to 2.3)	<0.001	-	-
Wealth quintile												
Poorest	(base)	-	-	-	(base)	-	-	-	(base)	-	-	-
Poor	1.02 (0.82 to 1.26)	0.86	-	-	0.92 (0.73 to 1.15)	0.46	-	-	0.93 (0.73 to 1.20)	0.59	-	-
Middle	0.97 (0.79 to 1.20)	0.79	-	-	0.94 (0.75 to 1.18)	0.60	-	-	1.20 (0.93 to 1.56)	0.16	-	-
Rich	1.11 (0.89 to 1.37)	0.36	-	-	1.32 (1.06 to 1.65)	0.01	-	-	1.55 (1.18 to 2.04)	0.002	-	-
Richest	0.76 (0.60 to 0.96)	0.02	-	-	1.10 (0.86 to 1.40)	0.44	-	-	1.61 (1.19 to 2.17)	0.002	-	-
*Level 2: Intermediary factors—household and personal characteristics*												
Marital status												
Married	(base)	-	-	-	(base)	-	-	-	(base)	-	-	-
Unmarried	1.04 (0.84 to 1.29)	0.73	-	-	1.24 (0.98 to 1.56)	0.075	-	-	1.46 (1.22 to 1.75)	<0.001	-	-
Has been pregnant												
Yes	(base)	-	-	-	(base)	-	-	-	(base)	-	-	-
No	1.07 (0.79 to 1.45)	0.65	-	-	1.24 (0.90 to 1.72)	0.19	-	-	1.12 (0.86 to 1.45)	0.41	-	-
Household food security status												
Food insecure	(base)	-	-	-	(base)	-	-	-	(base)	-	-	-
Food secure	0.88 (0.76 to 1.02)	0.09	-	-	1.18 (1.01 to 1.38)	0.04	-	-	1.13 (0.79 to 1.62)	0.50	-	-
*Level 3: Intermediary factors—health and well-being characteristics*												
Perception of own health												
Poor or fair	(base)	-	-	-	(base)	-	-	-	(base)	-	-	-
Good	0.81 (0.66 to 1.00)	0.05	-	-	1.14 (0.91 to 1.43)	0.25	-	-	1.56 (1.22 to 1.98)	<0.001	0.43 (0.16 to 0.71)	0.002
Excellent	0.87 (0.69 to 1.10)	0.25	-	-	1.27 (0.99 to 1.64)	0.06	-	-	1.50 (1.13 to 1.98)	0.004	0.32 (0 to 0.63)	0.05
Experience of depression-like feelings												
Severe or extremely severe	(base)	-	-	-	(base)	-	-	-	(base)	-	-	-
Moderate	1.06 (0.72 to 1.55)	0.77	-	-	1.92 (1.27 to 2.91)	0.002	-	-	0.96 (0.61 to 1.54)	0.88	-	-
Mild	0.93 (0.63 to 1.37)	0.71	-	-	1.55 (1.01 to 2.38)	0.04	-	-	0.81 (0.51 to 1.28)	0.36	-	-
None	0.96 (0.69 to 1.34)	0.82	-	-	1.35 (0.93 to 1.96)	0.11	-	-	1.12 (0.75 to 1.68)	0.58	-	-
Experience of anxiety-like feelings												
Severe or extremely severe	(base)	-	-	-	(base)	-	-	-	(base)	-	-	-
Moderate	0.97 (0.75 to 1.25)	0.80	-	-	1.11 (0.85 to 1.45)	0.44	-	-	0.72 (0.53 to 0.98)	0.04	-	-
Mild	1.05 (0.76 to 1.44)	0.76	-	-	0.92 (0.66 to 1.29)	0.63	-	-	1.14 (0.76 to 1.73)	0.53	-	-
None	0.95 (0.77 to 1.18)	0.64	-	-	1.00 (0.80 to 1.25)	0.98	-	-	1.01 (0.77 to 1.32)	0.95	-	-
Experience of stress-like feelings												
Severe or extremely severe	(base)	-	-	-	(base)	-	-	-	(base)	-	-	-
Moderate	1.06 (0.64 to 1.73)	0.83	-	-	0.96 (0.57 to 1.62)	0.89	-	-	2.55 (1.36 to 4.8)	0.004	-	-
Mild	1.11 (0.69 to 1.80)	0.67	-	-	0.94 (0.57 to 1.56)	0.81	-	-	1.45 (0.83 to 2.53)	0.19	-	-
None	1.11 (0.75 to 1.65)	0.60	-	-	1.10 (0.73 to 1.66)	0.64	-	-	1.39 (0.90 to 2.16)	0.14	-	-
*Level 4: Intermediary factors—actions and practices-related characteristics*												
Self-efficacy												
Low	(base)	-	-	-	(base)	-	-	-	(base)	-	-	-
Moderate	0.86 (0.74 to 1.00)	0.06	-	-	1.11 (0.94 to 1.30)	0.21	-	-	1.21 (1.00 to 1.46)	0.05	-	-
High	0.84 (0.69 to 1.03)	0.09	-	-	0.84 (0.67 to 1.04)	0.11	-	-	1.10 (0.86 to 1.40)	0.47	-	-
Decision-making autonomy												
All decisions made by family	(base)	-	-	-	(base)	-	-	-	(base)	-	-	-
Most decisions made by family	0.85 (0.73 to 0.99)	0.03	-	-	1.22 (1.05 to 1.43)	0.01	-	-	1.08 (0.89 to 1.31)	0.44	-	-
Jointly made with family or autonomously	0.84 (0.68 to 1.03)	0.10	-	-	0.98 (0.78 to 1.22)	0.85	-	-	0.76 (0.59 to 0.97)	0.04	-	-
Skipping breakfast												
Skips breakfast	(base)	-	-	-	(base)	-	-	-	(base)	-	-	-
Eats breakfast	1.00 (0.86 to 1.16)	0.97	-	-	0.95 (0.81 to 1.11)	0.50	-	-	1.11 (0.92 to 1.33)	0.29	-	-
Eating dinner with family												
Never	(base)		-	-	(base)	-	-	-	(base)		-	-
Sometimes	1.17 (0.92 to 1.50)	0.20	-	-	0.75 (0.57 to 0.98)	0.04	-	-	0.76 (0.57 to 1.02)	0.07	-	-
Everyday	1.09 (0.93 to 1.29)	0.29	-	-	1.18 (0.99 to 1.39)	0.06	-	-	1.02 (0.83 to 1.25)	0.83	-	-

## Data Availability

The anonymized, individual-level data from this cross-sectional assessment, situated within a clinical trial, will be made available upon reasonable request from the corresponding author.

## Supplementary Materials

The following are available online at https://www.mdpi.com/article/10.3390/nu13041237/s1, Table S1. Characteristics of biomarker assays used for determining micronutrient status. Table S2. Description of SDoH explanatory variables by hierarchical level.
