# Supplementary material for: Characterizing Micronutrient Status and Risk Factors among Late Adolescent and Young Women in Rural Pakistan: A Cross-Sectional Assessment of the MaPPS Trial
Source: Nutrients. 2021 Apr 9;13(4):1237. doi: 10.3390/nu13041237 (PMC8069550; doi:10.3390/nu13041237)
Supplement: Supplementary file 1 [file nutrients-13-01237-s001.pdf]

Supplemental Material

Table S1. Characteristics of biomarker assays used for determining micronutrient status.

Assay	Methodology	Analyzer	Kit used	Inter-assay CV (%)	Duplicate measurements n (%)	Standard reference material	Quality control methods
Ferritin	Particle enhanced immunoturbidimetric assay based on the immunological agglutination principle. Human ferritin agglutinates with latex particles coated with anti-ferritin antibodies. Precipitate determined turbidimetrically at 570/800 nm.	Roche Cobas C-311 automatic clinical chemistry analyzer	FERR4 (Tina-quant Ferritin Gen4), #04885317 190	4.36	394/12,033 (3.3)	Roche Elecsys Ferritin assay (immunological method) traceable to NIBSC (WHO)	Roche PreciControl ClinChem Multi-levels 1 and 2 (#05947626 190 and #05947774 190) used with each batch
Vitamin A	Retinol concentration determined by isocratic reversed-phase HPLC with UV/V detection at 325 nm using ODS column by internal standard method using retinyl acetate.	Agilent HPLC 1200 series	No kit used (Manual method)	6.47	138/11,861 (1.2)	Sigma Retinol (95144)	Third party ClinChek® Serum Control, lyophil., for Vitamins, Level I, II, III used (2 levels/batch)
Vitamin D	25-OH total vitamin D determined by auto immunoanalyzer using a direct competitive chemiluminescence immunoassay.	Diasorin Liaison automatic immunoanalyzer	Liaison 25 OH Vitamin D TOTAL assay kit, #310600	9.84	297/12,362 (2.4)	Not available	Quality control of the assay monitored internally with every batch of the samples using 25-OH Vitamin D TOTAL Control Set provided along with assay kit
CRP	CRP quantification in human serum/plasma is based on particle-enhanced immunoturbidimetric assay. Human CRP agglutinates with latex particles coated with monoclonal anti-CRP antibodies. The precipitate is determined turbidimetrically.	Roche Cobas C-311 automatic clinical chemistry analyzer	CRPLX (C-Reactive Protein [Latex]), #20764930 322	2.05	1620/12,025 (13.5)	Standardized against the reference preparation of the Institute for Reference Materials and Measurements (BCR470/CRM470, Reference Preparation for Proteins in Human Serum)	Roche PreciControl ClinChem Multi levels 1 and 2 (#05947626 190, #05947774 190) were used with each batch

Table S2. Description of SDoH explanatory variables by hierarchical level.

Variable	Tool/question source	Method of aggregation	Hypothesized relevance [a]
Level 1 – Structural SDoH - Socioeconomic status			
Education	Pakistan Demographic Health Survey [1]	- Categorization of highest completed grade<br>- Possible options: no education, some primary education, some secondary education or higher<br>- Base: no education	- Variable relates to socioeconomic position and improved resourcefulness<br>- Hypothesized that those with no education would be more nutritionally vulnerable
Occupation	Pakistan Demographic Health Survey [1]	- Categorization of reported occupations<br>- Possible options: within the home, unskilled manual labour, skilled manual labour, other (student or professional)<br>- Base: unskilled manual worker	- Variable serves as a measure of income earning<br>- Hypothesized that those who must perform unskilled manual labour would be more nutritionally vulnerable
Religion	Pakistan Demographic Health Survey [1]	- Possible options: Muslim, non-Muslim<br>- Base: non-Muslim (i.e., Hindu or Christian)	- Variable serves as a proxy for ethnicity, given existence of caste system within the study area<br>- Hypothesized that those who are of lower caste (non-Muslim) would be more nutritionally vulnerable
Wealth quintile [b]	Multiple questions from Pakistan Demographic Health Survey [1]	- Derived using a principal component analysis, from factors related to home characteristics and household asset ownership<br>- Base: poorest wealth quintile	- Variable indicative of socioeconomic class<br>- Hypothesized that those in lower quintiles would be more nutritionally vulnerable
Level 2 – Intermediary SDoH - Personal and household factors			
Household food security	Household Food Insecurity Access Scale [2]	- Recall period of past 30 days<br>- Experience-based food insecurity scale that captures households' behavioral and psychological manifestations of insecure food access<br>- Includes 9 occurrence and frequency of occurrence questions, which were used to categorize participants as food secure or insecure (includes mild, moderate, or severe)<br>- Base: food insecure	- Variable a household characteristic indicative of material circumstances (i.e., access to food)<br>- Hypothesized that those who are food insecure would be more nutritionally vulnerable
Marital status	Pakistan Demographic Health Survey [1]	- Categorical variable for whether currently married or not<br>- Base: married	- Variable a personal characteristic that could reflect social class and psychosocial circumstances (empowerment)<br>- Hypothesized that those who are married could be more nutritionally vulnerable
Parity	Pakistan Demographic Health Survey [1]	- Categorical variable for whether had or had not been pregnant before<br>- Base: has had a previous pregnancy	- Variable a personal characteristic that could reflect social class and psychosocial circumstances (empowerment)<br>- Hypothesized that those who have been pregnant could be more nutritionally vulnerable
Level 3 – Intermediary SDoH - Health and well-being			

Variable	Tool/question source	Method of aggregation	Hypothesized relevance [a]
Perception of own health	Health Behaviour in School-aged Children Questionnaire [3]	- Categorical variable of one's perceived health status<br>- Possible options: poor or fair, good, excellent<br>- Base: poor or fair	- Variable that reflects biological factors, albeit subjectively (health-related factors)<br>- Hypothesized that those who perceive their health to be less optimal could be more nutritionally vulnerable
Experience of depression-like feelings	DASS-21 assessment tool [4]	- Recall period: over last week<br>- Categorized from 7 questions within standardized tool, as outlined by authors of scale<br>- Severe and extremely severe categories were combined given the lack of observations (options: none, mild, moderate, severe, or extremely severe)<br>- Not a clinical diagnostic tool, rather allows for the measure of one's current emotional state over the reference period<br>- Base: severe or extremely severe	- Variable that could reflect biological factors and psychosocial circumstances (mental health)<br>- Hypothesized that those who report experiencing more depression-like feelings could be more nutritionally vulnerable
Experience of anxiety-like feelings	DASS-21 assessment tool [4]	- Same as above	- Variable that could reflect biological factors and psychosocial circumstances (mental health)<br>- Hypothesized that those who report experiencing more anxiety-like feelings could be more nutritionally vulnerable
Experience of stress-like feelings	DASS-21 assessment tool [4]	- Same as above	- Variable that could reflect biological factors and psychosocial circumstances (mental health)<br>- Hypothesized that those who report experiencing more stress-like feelings could be more nutritionally vulnerable
Level 4 – Intermediary SDoH - Actions and practices			
Self-efficacy	Schwarzer and Jerusalem's scale for self-efficacy [5]	- Based on level of agreement with ten statements about one's belief in their ability to execute a behaviour<br>- After scoring participants' responses, generated tertiles based on distribution<br>- Tertiles categorized as low, moderate, and high self-efficacy<br>- Base: low self-efficacy	- Variable reflects behavioural factors (empowerment)<br>- Hypothesized that those who report lower self-efficacy could be more nutritionally vulnerable

Variable	Tool/question source	Method of aggregation	Hypothesized relevance [a]
Participation in decision-making	Adapted for Pakistan Demographic Health Survey [1]	- Based on 5 factors pertaining to who makes decisions about: foods the participant eats, food distribution in the household, foods purchased in the household, household purchases for daily needs, participant's health care<br>- Participants list all people who made the decision<br>- Using on the five factors, categorized decision-making as all decisions made by family (reported contributing to no decisions), most decisions made by family (reported contributing to ≤30% of decisions), decisions made jointly with family or autonomously by participant (>30% decision contribution)<br>- Base: all decisions made by family	- Variable reflects behavioural factors (empowerment)<br>- Hypothesized that those who report family makes decisions could be more nutritionally vulnerable
Skipping breakfast	Health Behaviour in School-aged Children Questionnaire [2]	- Categorical variable for whether did or did not eat breakfast on a regular basis<br>- Base: skipped breakfast	- Variable reflects behavioural factors (food consumption)<br>- Hypothesized that those who report skipping breakfast could be more nutritionally vulnerable
Eating dinner with family	Health Behaviour in School-aged Children Questionnaire [2]	- Recall period: over past week<br>- Categorical variable for frequency of eating dinner with family: never (0 days), sometimes (1-6 days), everyday (7 days)<br>- Base: never eats dinner with family	- Variable reflects behavioural factors (food consumption)<br>- Hypothesized that those who report eating infrequent meals with family could be more nutritionally vulnerable

[a] Variable SDoH-related categorization guided by Fatsui and Bello 2015 [6].

[b] Because key factors related to water, sanitation, and hygiene were included in generating the variable for wealth index, or were highly homogeneous (i.e., handwashing practices), individual WASH-related variables were not considered within the modelling process.

References

1. National Institute of Population Studies (NIPS) [Pakistan] and ICF International. Pakistan Demographic and Health Survey 2012–13. Islamabad and Calverton: NIPS and ICF International; 2013.
2. Coates J, Swindale A, Bilinsky P. Household food insecurity access scale (HFIAS) for measurement of household food access: Indicator guide (v. 3). Washington: Food and Nutrition Technical Assistance Project, Academy for Educational Development; 2007.
3. Currie C, Inchley J, Molcho M, Lenzi M, Veselska Z, Wild F. Health Behaviour in School-aged Children (HBSC) Study Protocol: Background, Methodology, and Mandatory items for the 2013/14 Survey. St. Andrews: CAHRU; 2014.
4. Lovibond SH, Lovibond PF. Manual for the Depression Anxiety Stress Scales. Sydney: Psychology Foundation; 1995.
5. Schwarzer R, Jerusalem M. Generalized self-efficacy scale. In: Weinman J, Wright S, Johnston M, editors. Measures in health psychology: a user's portfolio. Causal and control beliefs. Windsor: NFER-NELSON; 1995. p. 35–7.
6. Fatusi AO, Bello B. Social Determinants of Health in Adolescents and Young Women's Health and Nutrition: Current Evidence. In Bhutta ZA, Makrides M, Prentice AM (Eds.), Health and nutrition in adolescents and young women: Preparing for the next generation (Nestlé Nutrition Institute Workshop Series, Vol. 80, pp. 61–69). Switzerland: S. Karger AG; 2015.
